# Development and Validation of an Mesenchymal-Related Long Non-Coding RNA Prognostic Model in Glioma

**DOI:** 10.3389/fonc.2021.726745

**Published:** 2021-09-03

**Authors:** Kebing Huang, Xiaoyu Yue, Yinfei Zheng, Zhengwei Zhang, Meng Cheng, Lianxin Li, Zhigang Chen, Zhihao Yang, Erbao Bian, Bing Zhao

**Affiliations:** ^1^Department of Neurosurgery, The Second Affiliated Hospital of Anhui Medical University, Hefei, China; ^2^Cerebral Vascular Disease Research Center, Anhui Medical University, Hefei, China

**Keywords:** mesenchymal, lncRNA, prognosis, glioma, TCGA, CGGA, immune

## Abstract

Glioma is well known as the most aggressive and prevalent primary malignant tumor in the central nervous system. Molecular subtypes and prognosis biomarkers remain a promising research area of gliomas. Notably, the aberrant expression of mesenchymal (MES) subtype related long non-coding RNAs (lncRNAs) is significantly associated with the prognosis of glioma patients. In this study, MES-related genes were obtained from The Cancer Genome Atlas (TCGA) and the Ivy Glioblastoma Atlas Project (Ivy GAP) data sets of glioma, and MES-related lncRNAs were acquired by performing co-expression analysis of these genes. Next, Cox regression analysis was used to establish a prognostic model, that integrated ten MES-related lncRNAs. Glioma patients in TCGA were divided into high-risk and low-risk groups based on the median risk score; compared with the low-risk groups, patients in the high-risk group had shorter survival times. Additionally, we measured the specificity and sensitivity of our model with the ROC curve. Univariate and multivariate Cox analyses showed that the prognostic model was an independent prognostic factor for glioma. To verify the predictive power of these candidate lncRNAs, the corresponding RNA-seq data were downloaded from the Chinese Glioma Genome Atlas (CGGA), and similar results were obtained. Next, we performed the immune cell infiltration profile of patients between two risk groups, and gene set enrichment analysis (GSEA) was performed to detect functional annotation. Finally, the protective factors DGCR10 and HAR1B, and risk factor SNHG18 were selected for functional verification. Knockdown of DGCR10 and HAR1B promoted, whereas knockdown of SNHG18 inhibited the migration and invasion of gliomas. Collectively, we successfully constructed a prognostic model based on a ten MES-related lncRNAs signature, which provides a novel target for predicting the prognosis for glioma patients.

## Introduction

Glioma accounts for 40% of intracranial tumors, which is the most common primary malignant tumor in the central nervous system (CNS) (1). According to the World Health Organization (WHO) classification, gliomas are divided into four different grades (grades I~IV). Among them, WHO II and WHO III are classified as lower grade glioma (LGG) and WHO IV as glioblastoma (GBM), which is the most aggressive type of brain tumor; neo-angiogenesis and invasion are the hallmarks of GBM (2, 3). Despite the existence of surgery, supplemented by chemotherapy, radiotherapy, and other treatment methods, the median survival of GBM patients is approximately 15 months (4, 5), and the 5-year survival rate is below 10% (6, 7). Additionally, based on differences in gene expression, TCGA classifies GBMs into classical, mesenchymal, neural, and proneural subtypes (8). Notably, approximately 45% of GBM tissues have been classified as the mesenchymal subtype, which is particularly malignant as compared to the other subtypes. The mesenchymal subtype is dominated in the relapses of GBM, and it has been revealed that cells of this subtype may have a higher therapy resistance (9). The overexpression of mesenchymal subtype (MES) related genes is adequate to induce invasive behavior in tumors and result in poor prognosis in patients (10).

LncRNA is a type of RNA that is longer than 200 nucleotides and lacks protein-coding ability (11). However, they are identified as having multiple biological functions, including the regulation of transcription, splicing, and translation (12). The biological function and carcinogenic mechanism of lncRNAs have been widely explored. Increasing evidence suggests that abnormal lncRNA expression has important significance in tumorigenesis and aggressiveness. For example, lncRNA ROR1-AS1 promotes glioma progression by inhibiting miR-4686 (12). The expression level of NEAT1 significantly increases in glioma tissues and promoted cell migration and invasion by regulating the miR‐139‐5p/CDK6 pathway (13). MES-related lncRNA miR155HG binds to miR-185 to affect proliferation, cell cycle progression, and apoptosis in GBM cell lines (14). MES-related lncRNA FAM181A-AS1 promotes the growth and survival of glioma cells by enhancing ZRANB2 expression (15). The mesenchymal subtype is characterized by higher percentages of cycling cells and neo-angiogenesis, with a highly invasive nature and poor prognosis (16). Furthermore, biological function analysis revealed that immune checkpoint receptor target was highly enriched in mesenchymal subtype glioma and might be a potential marker of mesenchymal subtype (17, 18).

Although the WHO classification system has been used to predict the prognosis of glioma patients for many years, it is sometimes inaccurate considering the heterogeneity of the tumor. New advances in bioinformatics and genome sequencing technology have helped to predict the prognosis of cancer patients in addition to identifying potential biomarkers (18, 19). Studies have shown that the prognostic value of a single candidate lncRNA biomarker is limited, integrating multiple biomarkers into a single model would be much better (20). For example, based on the metastasis-associated competing endogenous RNA (ceRNA) network, three lncRNAs were confirmed to have the ability to predict colorectal cancer (CRC) prognosis (21). By mining the TCGA data, a four-lncRNA signature could effectively predict the survival time of lung adenocarcinoma (LUAD) (22). Recently, some lncRNA prognostic models have been constructed, and their ability to predict have been validated in glioma. For example, an immune-related lncRNA formula provides a powerful prognostic prediction ability for glioma patients; similarly, ten autophagy-related lncRNAs have prognostic potential for glioma (23, 24).

Therefore, we speculated that identification of MES-related lncRNAs act as prognostic models is of great significance for discovery of prognostic biomarkers, evaluating therapeutic effect and development of more accurate treatment processes. In this study, we first analyzed the MES-related mRNA expression profile data in the TCGA and Ivy GAY databases. Then, the corresponding lncRNAs were obtained through co-expression analysis, and differentially expressed lncRNAs were identified between LGG and GBM samples. Next, MES-related lncRNAs with prognostic value based on Cox analysis were screened. Ultimately, we identified that a ten-lncRNA signature acts as an independent predictive factor for prognosis prediction in glioma patients. Based on the median risk score, glioma patients in the TCGA and CGGA databases were divided into high- and low-risk groups, and the results of the immune cell infiltration profile and GSEA revealed that the high-risk group was closely related to the tumor immune microenvironment and many aspects of glioma progression compared to the low-risk group. In addition, functional experiments further reveal the biological characteristics of glioma cell lines, which will be helpful in advancing the development of targeted treatment in glioma.

## Materials and Methods

### Data Acquisition

The RNA-seq data of MES-related genes were downloaded from TCGA (https://cancergenome.nih.gov/) and Ivy GAP (http://glioblastoma.alleninstitute.org/). Ivy GAP is a freely accessible online data resource for exploring the anatomic and genetic basis of glioblastoma at the cellular and molecular levels, including digitized tissue pathology slides, and corresponding transcriptomic data of GBM patients (25, 26).

In TCGA, clinical information included gender, cancer type and Karnofsky Performance Score (KPS) score, etc. After 11 patients with incomplete clinical information were excluded, the training set included 666 samples from TCGA. In order to further validated the accuracy of the results, the testing set included 618 samples from the Chinese Glioma Genome Atlas (CGGA, http://www.cgga.org.cn/, freely available) dataset, the clinical information included primary-recurrent-secondary (PRS) type, grade, gender, age, radio status, chemo status, IDH mutation status, 1p19q codeletion status, etc.

### Identification of MES-Related LncRNAs

We obtained 303 MES-related encoding genes (mRNAs) from two datasets (TCGA and Ivy GAP). Then, 47 MES-related lncRNAs were identified by constructing MES-related mRNA-lncRNA co-expression network according to the criteria of |Correlation Coefficient| > 0.7 and P <0.001 by Pearson correlation analysis using the Limma R package. Next, we carried out a difference analysis between LGG and GBM samples with the R programming language (http://cran.r-project.org) and 42 differentially expressed lncRNAs were identified.

### Construction of a Prognostic Model With MES-Related LncRNA

In order to narrow the scope, univariate and multivariate Cox regression was performed in the TCGA data set, and ultimately, 10 MES-related lncRNAs were used as candidates for the prognostic model. HR<1 was considered a protective factor, whereas HR>1 was considered a risk factor. In order to compute the risk score of each glioma patient, multivariate regression analysis was performed to evaluate the relative contribution of candidate lncRNAs as prognostic models. The formula was as follows:

$$\text{Risk Score}=\Sigma_{i=1}^{n}\;coef(i)\times x(i)$$

Coef (i) and X(i) represent the regression coefficient and expression value of MES-related lncRNAs, respectively.

### Evaluation of the Prognostic Model

Using the median risk score as the demarcation point, glioma patients were divided into high-risk and low-risk groups. Kaplan-Meier survival curves were used to compare the OS of the two groups of glioma patients. In order to determine whether the risk score model is an independent factor for glioma patients, we performed univariate and multivariate Cox regression analysis of these prognostic factors, and the ROC curves were used to assess the predictive value of the prognostic model. P<0.05 was considered statistically significant.

### Estimation of the Immune Cell Composition and Bioinformatics Analysis

The single sample gene set enrichment analysis (ssGSEA) was carried out to explore the different infiltration degrees of 24 immune cell types in two kind risk groups with the R package “GSV A”. We evaluate tumor purity by the R package “ESTIMA TE,” which is based on the estimation of stromal and immune cell markers (27). PCA was performed with R software to explore the expression patterns between low- and high-risk groups based on the ten MES-related lncRNAs. GSEA software (4.0.1) (http://www.broadinstitute.org/gsea/index.jsp) was used for gene set enrichment analysis to discern differences in gene sets between the low- and high-risk groups. To validate these lncRNA expression levels, the Gene Expression Profiling Interactive Analysis (GEPIA, http://gepia.cancer-pku.cn/) was applied to analyze the RNA sequencing data.

### Cell Culture and Transfection

The glioma cell lines LN18, SNB19, SW1088, T98G, and U251 were purchased from Shanghai Institute of Cell Biology, Chinese Academy of Sciences (Shanghai, China). All cell lines were cultured in Dulbecco’s Modified Eagle’s medium (DMEM : SH30022.01; HyClone) containing with 10% fetal bovine serum (FBS, Gibco) at the culture condition of 37°C with 5% CO2 in a humidified incubator. The cells grew in a monolayer, with the medium needing to be changed every 48 hours. Then, we used lncRNA Smart Silencer, antisense oligonucleotides (ASOs), and small interfering RNAs (siRNAs), designed by RIBOBIO (Guangzhou Ribobio Co.), to target and knockdown the expression of DGCR10, HAR1B, and SNHG18. The sequences were as follows: DGCR5 Smart Silencer, CCTTCACTCTGGTCATCGTT; ASO-HAR1B, CAACACTTGAACAAGCAAGG; si-SNHG18, CCACTTGGATTTCACCAAA. Next, in accordance with the manufacturer’s instructions in Opti-MEM (Gibco; Thermo Fisher Scientific, Inc.), transfection reagent jetPRIME (Poly plus-transfection^®^) was used to carry out cell transfection.

### RNA Extraction and RT-qPCR

TRIzol reagent (Invitrogen, Thermo Fisher Scientific) was used to extract total RNA from LN18, U251, and T98G cells according to the manufacturer’s protocol. Reverse transcription was performed using PrimeScript RT Master Mix (Perfect Real Time) (RR036A; Takara). Next, RT-qPCR was carried out with TB Green™ Premix Ex Taq™ II (Tli RNaseH Plus) (RR820A; Takara); the steps for RT-qPCR reaction were as follows: predenaturation at 95°C, 30s, one cycle; quantitative analysis at 95°C for 5s, 60°C for 31s, 40 cycles; dissolution curve at 95°C for 15s, 60°C for 1min, 95°C for 15s, 1 cycle. The relative expression level of each lncRNA was calculated using the 2−ΔΔCt method. The primer sequences of GAPDH, DGCR10, HAR1B, and SNHG18 are shown in **Table 1**.

**Table 1:** The primer sequences of DGCR10, HAR1B, and SNHG18.

GAPDH	F primer(5’-3’)	CGCTCTCTGCTCCTCCTGT
	R primer(5’-3’)	ATCCGTTGACTCCGACCTA
DGCR10	F primer(5’-3’)	TGTTTCAGAAGCACCGTCAG
	R primer(5’-3’)	CCCTCACTTGAATGGATGCT
HAR1B	F primer(5’-3’)	CCTGGGGCTAAATGAATGAA
	R primer(5’-3’)	GTTGAGTGAGGGCAGTCTCC
SNHG18	F primer(5’-3’)	GTTGCACTTTGCCACTGCTA
	R primer(5’-3’)	GGAATGTGGTTCTCCCTTGA

F primer, forward primer; R primer, reverse primer.

### Transwell Migration Assay and Matrigel Invasion Assay

First, transwell migration assay were performed to measure LN18, U251, and T98G cell migration ability. Transfected (2×10^4^) cells were added into the upper chamber that contained 200 µl of serum-free DMEM. Meanwhile, the lower chamber contained 600 µl of DMEM with 30% FBS and was cultured at 37°C for 24h. For the invasion assay, 1×10^5^ cells were plated in each chamber that was pre-coated with Matrigel (356234; BD Biosciences) and cultivated for 48h in a culture environment of 37°C and 5% CO2. After incubation, 4% polyoxymethylene and 0.5% crystal violet were used to fix and stain the cells, respectively. Then, a cotton swab was used to remove cells remaining on the upper surface of the parietal chamber. Quantify under a microscope and perform three independent experiments.

### Statistical Analysis

GraphPad Prism 8 was used for statistical analysis. A co-expression network of the 47 MES-related mRNA-lncRNA was established and visualized using Cytoscape software(version 3.4.0) (28). ImageJ software for Microsoft Windows was used for the cell number counts. The univariate and multivariate Cox regression analyses were carried out using R version 3.6.2 and relevant packages. Perl version 5.30.2 (http://www.perl.org) was used to process above data.

Statistical significance was denoted as follows: *P < 0.05, **P < 0.01, ***P < 0.001.

## Results

### Identification and Differential Expression Profiles of MES-Related LncRNAs

**Figure 1:**
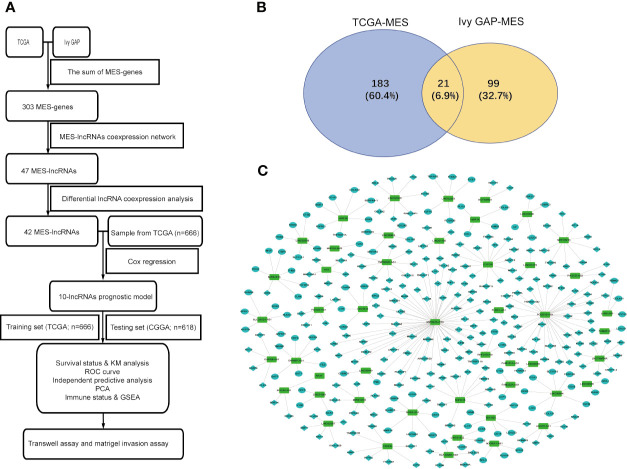
Identification of MES-related lncRNAs. **(A)** Flowchart of our study. **(B)** Venn diagram: the sum aggregate of MES-related mRNAs in the TCGA and Ivy GAP data sets. **(C)** Network of MES-related lncRNAs with co-expressed MES-related genes in glioma. In the centric position, green nodes indicate MES-related lncRNAs and the sky blue indicates MES-related genes, diamonds indicate positive correlation, ellipses indicate negative correlation. The coexpression network is visualized by CYTOSCAPE 3.4 software.

**Figure 2:**
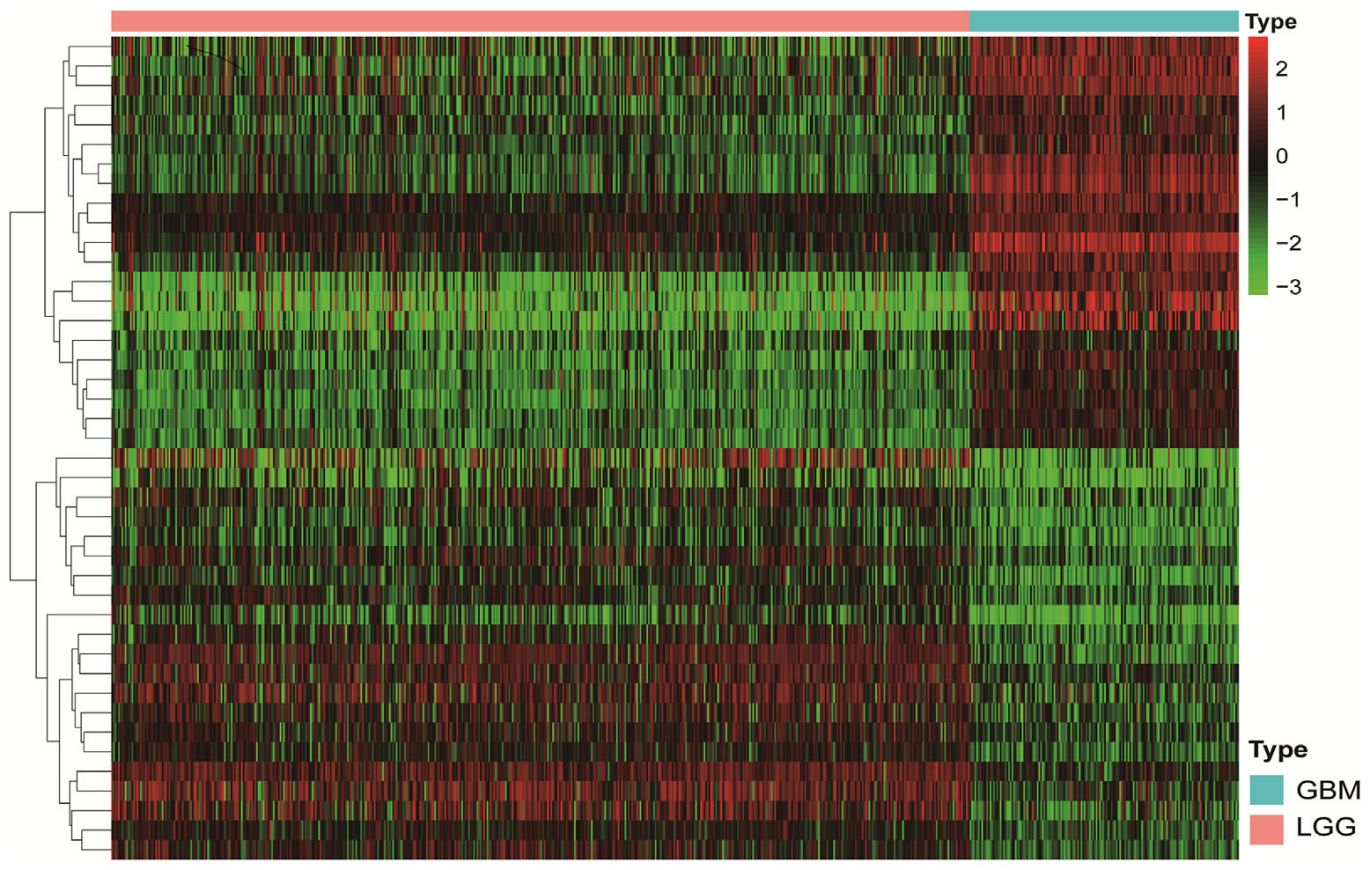
The Heatmap shows that 42 MES-related lncRNAs with obvious discrepancies between LGG and HGG. The color from green to red shows an increasing trend from low levels to high levels.

A flowchart describing the construction and verification of our prognostic model was first drafted (**Figure 1A**). The MES-related mRNAs were obtained from the TCGA and Ivy GAP databases, with 204 MES-related mRNAs from TCGA and 120 from Ivy GAP. The Venn diagram shows a total of 303 MES-related mRNAs contained in the two databases (**Figure 1B**). Next, A total of 47 MES-related lncRNAs were obtained by constructing the co-expression networks with 303 MES-related mRNAs (**Figure 1C** and **Table 2**). Then, 42 differentially expressed lncRNAs were identified between the LGG samples and GBM samples in the TCGA database (**Figure 2**). In order to narrow the scope, 18 MES-related lncRNAs were screened by performing univariate Cox regression analysis (**Figure S1A**). Multivariate Cox regression analysis showed that 10 MES-related lncRNAs (GDNF-AS1, CRNDE, FAM201A, HAR1B, AGAP2-AS1, RNF219-AS1, DGCR10, SNHG18, LINC00906, HAR1A) were significantly associated with prognosis (**Figure S1B**), out of which three lncRNAs were unfavorable (CRNDE, AGAP2-AS1, and SNHG18) and the remaining seven were favorable factors.

**Table 2:** Correlation between the MES-related lncRNAs and MES-related genes in glioma.

MES-gene	lncRNA	Correlation	Pvalue	Regulation
BCL3	ADGRA1-AS1	-0.723251249	1.68E-109	negative
SP100	ADGRA1-AS1	-0.737913942	3.55E-116	negative
PLAUR	ADGRA1-AS1	-0.73035445	1.12E-112	negative
EMP3	AGAP2-AS1	0.731906206	2.18E-113	postive
RAB34	AGAP2-AS1	0.715618947	3.42E-106	postive
EFEMP2	AGAP2-AS1	0.703334457	4.29E-101	postive
PDPN	AGAP2-AS1	0.75489171	1.72E-124	postive
TIMP1	AGAP2-AS1	0.720369625	3.08E-108	postive
PLK3	CARD8-AS1	0.704190201	1.93E-101	postive
SCPEP1	CARD8-AS1	0.742354745	2.75E-118	postive
CASP4	CARD8-AS1	0.848233137	1.48E-186	postive
RAC2	CARD8-AS1	0.76374507	4.21E-129	postive
LCP2	CRNDE	0.767587393	3.63E-131	postive
SIGLEC7	CRNDE	0.722199506	4.88E-109	postive
FCGR2A	CRNDE	0.790736168	1.64E-144	postive
TNFRSF1B	CRNDE	0.739270954	8.13E-117	postive
TRIM38	CRNDE	0.734919647	8.91E-115	postive
PTPRC	CRNDE	0.703111811	5.28E-101	postive
ESM1	CRNDE	0.808731041	4.11E-156	postive
ANXA1	CYTOR	0.768664703	9.41E-132	postive
ZYX	CYTOR	0.704194157	1.93E-101	postive
TES	CYTOR	0.822364252	9.32E-166	postive
S100A4	CYTOR	0.728910892	5.03E-112	postive
RAB32	CYTOR	0.789578834	8.36E-144	postive
ANGPT2	CYTOR	0.751804533	6.28E-123	postive
CHI3L1	CYTOR	0.841494355	8.83E-181	postive
ADAM12	CYTOR	0.801655282	2.08E-151	postive
HSD3B7	CYTOR	0.777152401	1.74E-136	postive
FAM20C	CYTOR	0.728020205	1.27E-111	postive
RABGAP1L	CYTOR	0.817769613	2.04E-162	postive
MGAT1	CYTOR	0.824710651	1.68E-167	postive
HFE	CYTOR	0.881760834	3.55E-220	postive
NPC2	DGCR10	-0.75119787	1.27E-122	negative
C1R	DGCR10	-0.731074245	5.24E-113	negative
TCIRG1	DGCR10	-0.713549452	2.58E-105	negative
C1orf54	DGCR10	-0.729196078	3.74E-112	negative
ANXA2	DGCR10	-0.7418703	4.70E-118	negative
PYGL	DNMBP-AS1	0.700273705	7.29E-100	postive
NCF4	DNMBP-AS1	-0.720949302	1.72E-108	negative
BATF	DNMBP-AS1	-0.734240073	1.84E-114	negative
FES	DNMBP-AS1	-0.719778576	5.56E-108	negative
SAT1	DNMBP-AS1	-0.712540126	6.87E-105	negative
CECR2	DNMBP-AS1	-0.70918087	1.73E-103	negative
TMBIM1	DNMBP-AS1	-0.725498591	1.70E-110	negative
SWAP70	FAM181A-AS1	0.709042986	1.98E-103	postive
LGALS1	FAM201A	-0.721622653	8.74E-109	negative
LCP1	FAM222A-AS1	-0.792884013	7.79E-146	negative
CTSZ	FAM222A-AS1	-0.726745712	4.73E-111	negative
S100A11	FOXD3-AS1	0.792119816	2.31E-145	postive
LAPTM5	FOXD3-AS1	0.90175565	1.59E-245	postive
CASP1	FOXD3-AS1	0.821130902	7.51E-165	postive
VAMP5	FOXD3-AS1	0.741324059	8.57E-118	postive
ASL	FOXD3-AS1	0.736419271	1.78E-115	postive
CASP8	GDNF-AS1	0.701231718	3.02E-100	postive
MMP14	GDNF-AS1	-0.741238825	9.42E-118	negative
CSTA	GDNF-AS1	-0.701958292	1.54E-100	negative
CEBPB	GDNF-AS1	-0.751505331	8.88E-123	negative
ALOX5	GDNF-AS1	-0.701965706	1.53E-100	negative
MGST2	GDNF-AS1	-0.71173905	1.49E-104	negative
UNC93B1	GDNF-AS1	-0.722893754	2.42E-109	negative
SLAMF8	GDNF-AS1	-0.716235612	1.86E-106	negative
SRPX2	H19	0.797310338	1.30E-148	postive
SERPINA1	H19	0.764942099	9.68E-130	postive
SLC10A3	H19	0.742151142	3.44E-118	postive
TRADD	H19	0.854298278	5.33E-192	postive
GRN	H19	0.77322545	2.86E-134	postive
SHC1	HAR1A	-0.766667477	1.14E-130	negative
PLAU	HAR1A	-0.764539245	1.59E-129	negative
COL5A1	HAR1A	-0.714744644	8.04E-106	negative
LAMB1	HAR1A	-0.720832383	1.93E-108	negative
COL4A2	HAR1B	-0.78360979	3.15E-140	negative
TNFRSF1A	HAR1B	-0.798349091	2.83E-149	negative
MSR1	HAR1B	-0.745131067	1.25E-119	negative
DOK3	HAR1B	-0.735007986	8.11E-115	negative
SERPINH1	HAR1B	-0.71561448	3.43E-106	negative
CD151	HAR1B	-0.706972547	1.41E-102	negative
FGFRL1	HAR1B	-0.722729715	2.86E-109	negative
MS4A4A	HLA-DQB1-AS1	0.705436576	6.01E-102	postive
PTPN6	HLA-DQB1-AS1	0.891936216	1.88E-232	postive
FXYD5	HLA-DQB1-AS1	0.767059922	7.01E-131	postive
SIGLEC9	HLA-DQB1-AS1	0.778864005	1.83E-137	postive
UCP2	HLA-DQB1-AS1	0.703534982	3.56E-101	postive
IQGAP1	HOTAIRM1	0.703927818	2.47E-101	postive
CAST	HOTAIRM1	0.712165787	9.86E-105	postive
LGALS3	ISX-AS1	-0.715131654	5.51E-106	negative
CLCF1	ISX-AS1	-0.711788793	1.42E-104	negative
MYO1F	ISX-AS1	-0.784811149	6.14E-141	negative
CD14	ISX-AS1	-0.718910966	1.32E-107	negative
ACTN1	ISX-AS1	0.718192731	2.70E-107	postive
OSMR	ISX-AS1	-0.72350741	1.30E-109	negative
COL4A1	LHX5-AS1	-0.744960521	1.51E-119	negative
RBMS1	LHX5-AS1	-0.722094748	5.43E-109	negative
SOCS3	LHX5-AS1	-0.754238324	3.70E-124	negative
MAP2K3	LINC00463	-0.800421754	1.31E-150	negative
SERPINE1	LINC00463	-0.741280119	9.00E-118	negative
SEC24D	LINC00463	-0.730248934	1.25E-112	negative
COL1A2	LINC00463	-0.75052645	2.74E-122	negative
RUNX1	LINC00836	-0.708421807	3.58E-103	negative
ARSJ	LINC00836	-0.708790773	2.52E-103	negative
LOX	LINC00844	-0.70708702	1.27E-102	negative
ITGA5	LINC00844	-0.730485681	9.72E-113	negative
HEXB	LINC00844	-0.744550631	2.39E-119	negative
SLC16A3	LINC00844	-0.743016749	1.32E-118	negative
TGFBI	LINC00844	-0.713897087	1.84E-105	negative
COL1A1	LINC00844	-0.724914541	3.10E-110	negative
ITGA1	LINC00844	-0.703582656	3.41E-101	negative
FNDC3B	LINC00844	-0.701689348	1.98E-100	negative
LIF	LINC00906	-0.724510168	4.68E-110	negative
ZNF217	LINC00906	-0.784182605	1.45E-140	negative
OSBPL3	LINC00906	-0.707498089	8.60E-103	negative
PLA2G5	LINC00906	-0.713940109	1.76E-105	negative
LHFPL2	LINC01150	0.82591464	2.10E-168	postive
CLEC2B	LINC01150	0.731082239	5.20E-113	postive
CD4	LINC01150	0.741416973	7.74E-118	postive
RRAS	LINC01150	0.775058945	2.68E-135	postive
SYNGR2	LINC01532	-0.705471341	5.82E-102	negative
STXBP2	LINC01532	-0.748283615	3.56E-121	negative
CCR5	LINC01532	-0.725695514	1.39E-110	negative
NCF2	LINC01561	-0.794295627	1.03E-146	negative
DEF6	LINC01561	-0.701078216	3.48E-100	negative
LY96	LINC01561	-0.791651035	4.50E-145	negative
C5AR1	LINC01579	0.716187996	1.95E-106	postive
GNA15	LINC01579	0.71409515	1.52E-105	postive
RHOG	LINC02058	-0.705534286	5.49E-102	negative
SLC11A1	LINC02058	-0.706286364	2.70E-102	negative
CTSB	LINC02058	-0.702585144	8.62E-101	negative
EFNB2	LINC02058	-0.735097141	7.37E-115	negative
ITGB2	LINC02058	-0.768725875	8.71E-132	negative
FHOD1	LINC02283	-0.705970009	3.64E-102	negative
FCGR2B	LINC02283	-0.706014089	3.49E-102	negative
TNFAIP3	LINC02283	-0.705000341	9.06E-102	negative
ECE1	LINC02283	-0.707997246	5.35E-103	negative
KLF16	LINC02283	-0.720161694	3.79E-108	negative
COL8A2	LINC02308	0.768037977	2.07E-131	postive
IFITM2	LINC02308	0.866781293	5.03E-204	postive
CTSC	LINC02308	0.863165639	2.02E-200	postive
VDR	LINC02308	0.753456102	9.23E-124	postive
RELB	LINC02308	0.872315674	9.40E-210	postive
RAB11FIP1	LINC02308	0.711018372	2.98E-104	postive
PROCR	LINC02308	0.796816986	2.67E-148	postive
PML	LINC02440	-0.754426179	2.97E-124	negative
CYBRD1	LINC02440	-0.70405237	2.20E-101	negative
PI3	LINC02587	0.743298904	9.66E-119	postive
BLVRB	LINC02587	0.716151583	2.02E-106	postive
KIAA1429	LINC02587	0.792798264	8.81E-146	postive
EMP1	LINC02587	0.716079473	2.17E-106	postive
NRP1	LINC02587	0.734206645	1.91E-114	postive
HOMER3	LINC02593	-0.711446705	1.97E-104	negative
HRH1	LNCTAM34A	0.730068811	1.50E-112	postive
LAIR1	LNCTAM34A	0.729646902	2.34E-112	postive
TRIM22	LNCTAM34A	0.701211542	3.07E-100	postive
COPZ2	LNCTAM34A	0.712600892	6.47E-105	postive
NOD2	LNCTAM34A	0.712304444	8.63E-105	postive
UAP1	LNCTAM34A	0.719395984	8.14E-108	postive
ANGPTL4	LNCTAM34A	0.70382151	2.73E-101	postive
LTBP1	LNCTAM34A	0.732368484	1.34E-113	postive
TLR2	MIR124-2HG	-0.700312718	7.03E-100	negative
STAB1	MIR155HG	0.722595743	3.27E-109	postive
ARPC1B	MIR155HG	0.738460751	1.96E-116	postive
FBN1	MIR155HG	0.734528187	1.35E-114	postive
SGSH	MIR155HG	0.705974156	3.63E-102	postive
TNFAIP8	MIR155HG	0.760144672	3.34E-127	postive
ST14	MIR155HG	0.738271492	2.41E-116	postive
MYL9	MIR155HG	0.706645124	1.93E-102	postive
ITGAM	MIR155HG	0.760274296	2.86E-127	postive
TEC	MIR155HG	0.773521683	1.95E-134	postive
MRC2	MIR210HG	0.795149892	3.01E-147	postive
S100A13	MIR210HG	0.829105423	7.75E-171	postive
IL4R	MIR210HG	0.805398731	7.13E-154	postive
RUNX2	MIR210HG	0.772935587	4.15E-134	postive
PTPN22	MIR210HG	0.84141244	1.03E-180	postive
ACTA2	MIR210HG	0.786386666	7.06E-142	postive
KYNU	MIR4435-2HG	0.776398014	4.68E-136	postive
TGOLN2	MIR4435-2HG	0.812878293	5.80E-159	postive
P4HA2	MIR4435-2HG	0.731736263	2.61E-113	postive
POLD4	MIR4435-2HG	0.797478571	1.01E-148	postive
RBKS	MIR4435-2HG	0.759140903	1.12E-126	postive
ANPEP	MIR4435-2HG	0.822239751	1.15E-165	postive
WIPF1	MIR4435-2HG	0.800866984	6.76E-151	postive
ELF4	MIR4435-2HG	0.771481816	2.66E-133	postive
LILRB2	MIR4435-2HG	0.736658612	1.38E-115	postive
ITGA3	MIR4435-2HG	0.812569108	9.52E-159	postive
MAN1A1	MIR4435-2HG	0.836831098	6.12E-177	postive
MAFB	MIR4435-2HG	0.748383139	3.18E-121	postive
CA12	MIR4435-2HG	0.783922964	2.06E-140	postive
HEXA	MIR4435-2HG	0.723400705	1.45E-109	postive
SYPL1	MIR4435-2HG	0.713675544	2.28E-105	postive
LZTS1	MIR4435-2HG	0.79261516	1.14E-145	postive
ARHGAP29	MIR4435-2HG	0.834929266	2.08E-175	postive
STAT6	MIR4435-2HG	0.737255287	7.24E-116	postive
FHL2	MIR4435-2HG	0.813810352	1.30E-159	postive
MFSD1	MIR4435-2HG	0.739073012	1.01E-116	postive
LRRFIP1	MIR4435-2HG	0.731647538	2.87E-113	postive
GCNT1	MIR4435-2HG	0.740228347	2.86E-117	postive
DCBLD2	MIR4435-2HG	0.750334768	3.42E-122	postive
ACSL1	MIR4435-2HG	0.739373668	7.27E-117	postive
PLEKHF1	MIR4435-2HG	0.759259499	9.69E-127	postive
ITGA7	MIR4435-2HG	0.709824827	9.37E-104	postive
BDKRB2	MIR4435-2HG	0.798435123	2.49E-149	postive
JUNB	MIR4435-2HG	0.764253243	2.26E-129	postive
PTGER4	MIR4435-2HG	0.732071605	1.83E-113	postive
ICAM3	MIR4435-2HG	0.734477655	1.43E-114	postive
AMPD3	MIR4435-2HG	0.714384219	1.14E-105	postive
UGP2	MIR4435-2HG	0.757307838	9.94E-126	postive
DLC1	MIR4435-2HG	0.704999721	9.06E-102	postive
ACPP	MIR4435-2HG	0.819570192	1.03E-163	postive
DAB2	MIR4435-2HG	0.719984278	4.52E-108	postive
MYH9	MIR4435-2HG	0.700146587	8.20E-100	postive
THBS1	MIR4435-2HG	0.747407845	9.62E-121	postive
FMNL1	MIR4435-2HG	0.799316551	6.78E-150	postive
TRIM47	MIR4435-2HG	0.7222386	4.70E-109	postive
TNC	MIR4435-2HG	0.764180611	2.47E-129	postive
CASP5	MIR4435-2HG	0.837173036	3.23E-177	postive
IL1R1	MIR4435-2HG	0.719085775	1.11E-107	postive
SALL4	MIR4435-2HG	0.781681217	4.26E-139	postive
MAN2B1	MIR4435-2HG	0.71929111	9.04E-108	postive
C1RL	MIR4435-2HG	0.706475273	2.26E-102	postive
WWTR1	MIR4435-2HG	0.741034916	1.18E-117	postive
FLT1	MIR4435-2HG	0.713150876	3.80E-105	postive
NRP2	MIR4435-2HG	0.724429502	5.08E-110	postive
MAPK13	MIR4435-2HG	0.703625559	3.27E-101	postive
PHF11	MIR4435-2HG	0.79019623	3.51E-144	postive
TRIM56	MIR4435-2HG	0.802025431	1.19E-151	postive
MAN2A1	MIR4435-2HG	0.786706621	4.54E-142	postive
PLS3	MIR4435-2HG	0.767311168	5.12E-131	postive
B4GALT1	MIR4435-2HG	0.769066299	5.68E-132	postive
DSE	MIR4435-2HG	0.766587417	1.26E-130	postive
SLC39A8	MIR4435-2HG	0.808986476	2.75E-156	postive
ALDH3B1	MIR4435-2HG	0.866695373	6.14E-204	postive
ARAF	SOCS2-AS1	0.89099436	2.90E-231	postive
SPRY4	SOCS2-AS1	0.818257665	9.11E-163	postive
EDEM3	SOCS2-AS1	0.837284715	2.62E-177	postive
LTBP2	MIR9-3HG	-0.712240856	9.17E-105	negative
TGFBR2	MIR9-3HG	-0.72743999	2.31E-111	negative
GGN	MIR9-3HG	0.733151826	5.85E-114	postive
MVP	MIR9-3HG	-0.83583869	3.87E-176	negative
DSC2	MIR9-3HG	-0.724407928	5.20E-110	negative
TLR4	MIR9-3HG	-0.724246436	6.13E-110	negative
THBD	MIR9-3HG	-0.790971292	1.18E-144	negative
ENG	PCED1B-AS1	0.723371373	1.49E-109	postive
FOSL2	PCED1B-AS1	0.702507585	9.26E-101	postive
LILRB3	PCED1B-AS1	0.717062191	8.25E-107	postive
FURIN	PCED1B-AS1	0.70082055	4.41E-100	postive
BACE2	PCED1B-AS1	0.714396517	1.13E-105	postive
ICAM1	PCED1B-AS1	0.765958992	2.75E-130	postive
PAPPA	PCED1B-AS1	0.704269589	1.79E-101	postive
SH2B3	PCED1B-AS1	0.864760075	5.36E-202	postive
FGG	PCED1B-AS1	0.856117366	1.11E-193	postive
FOLR2	PCED1B-AS1	0.838224528	4.48E-178	postive
EHD2	PCED1B-AS1	0.711737081	1.49E-104	postive
ITGA4	PCED1B-AS1	0.711254985	2.38E-104	postive
EPAS1	PCED1B-AS1	0.793249908	4.62E-146	postive
PDGFA	PCED1B-AS1	0.727126202	3.20E-111	postive
CDCP1	PCED1B-AS1	0.833678947	2.06E-174	postive
CD2AP	PCED1B-AS1	0.92306236	2.23E-279	postive
TAGLN	PCED1B-AS1	0.711187785	2.53E-104	postive
C1QTNF1	PCED1B-AS1	0.836914503	5.23E-177	postive
TRPM2	PCED1B-AS1	0.848905907	3.79E-187	postive
YAP1	PCED1B-AS1	0.701301968	2.83E-100	postive
BNC2	PCED1B-AS1	0.806250958	1.93E-154	postive
PYGO2	PCED1B-AS1	0.850788541	8.06E-189	postive
TNFRSF10D	PCED1B-AS1	0.745288608	1.05E-119	postive
RRBP1	PCED1B-AS1	0.798978139	1.12E-149	postive
RAB27A	PCED1B-AS1	0.770905481	5.55E-133	postive
ANXA4	PCED1B-AS1	0.846256873	7.82E-185	postive
SLC12A9	PCED1B-AS1	0.816362784	2.06E-161	postive
LY75	PCED1B-AS1	0.747018761	1.49E-120	postive
FLNA	PCED1B-AS1	0.870700202	4.70E-208	postive
IGFBP6	PCED1B-AS1	0.752096749	4.48E-123	postive
IFITM3	PCED1B-AS1	0.775535902	1.44E-135	postive
PDGFRL	PCED1B-AS1	0.791857393	3.36E-145	postive
SFT2D2	PCED1B-AS1	0.718097667	2.96E-107	postive
IFI30	PIK3CD-AS2	0.735644783	4.10E-115	postive
CNN2	PIK3CD-AS2	0.807751526	1.89E-155	postive
RYR3	PIK3CD-AS2	0.739708812	5.04E-117	postive
HK3	PLBD1-AS1	0.735197544	6.62E-115	postive
TNFRSF11A	RNF219-AS1	-0.709996671	7.95E-104	negative
IL15RA	RNF219-AS1	-0.710121925	7.05E-104	negative
PTRF	RNF219-AS1	-0.731269659	4.27E-113	negative
NCSTN	SLC25A21-AS1	-0.716286662	1.77E-106	negative
NUCB1	SLC25A21-AS1	-0.723659314	1.11E-109	negative
SPRY1	SLC25A21-AS1	-0.831746912	6.88E-173	negative
GANAB	SLC25A21-AS1	-0.772856391	4.59E-134	negative
ARFRP1	SLC25A21-AS1	-0.828270791	3.39E-170	negative
SOX2	SLC25A21-AS1	-0.796417235	4.78E-148	negative
ADAM19	SNHG18	0.774459433	5.82E-135	postive
TM9SF4	SNHG18	0.715187227	5.22E-106	postive
TRIP10	SNHG18	0.70858243	3.07E-103	postive
KDELR1	SNHG18	0.704554996	1.37E-101	postive
FGFR1	SNHG18	0.723702946	1.07E-109	postive
TM9SF1	SNHG18	0.705392111	6.27E-102	postive
TRAF6	SNHG18	0.775102908	2.53E-135	postive
AP3B1	SNHG18	0.750317134	3.49E-122	postive
BAX	SNHG18	0.713940133	1.76E-105	postive
SPRED2	SNHG18	0.744691209	2.05E-119	postive
ARAF	TMEM220-AS1	0.779995802	4.06E-138	postive
PDGFR	TMEM220-AS1	0.730451083	1.01E-112	postive
BCAT1	TMEM220-AS1	0.725993195	1.03E-110	postive
CTGF	TMEM220-AS1	0.700062581	8.85E-100	postive
FOS	TMEM220-AS1	0.717566049	5.01E-107	postive
COLEC12	TMEM220-AS1	0.770718595	7.03E-133	postive
SFRP2	TMEM220-AS1	0.739682281	5.19E-117	postive
PDGFC	TMEM220-AS1	0.752499999	2.81E-123	postive

### Construction and Assessment of the Ten MES-Related LncRNAs Risk Score Model for Glioma Patients

**Figure 3:**
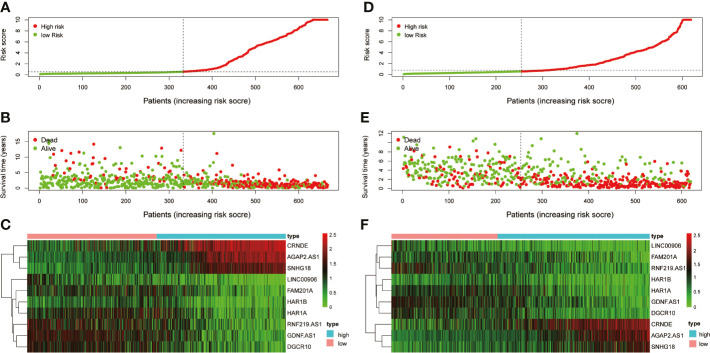
Examination and validation prognostic models based on candidate lncRNAs signatures. **(A)** Risk score distribution in the TCGA database. **(B)** Survival status and time of glioma patients in the TCGA database. **(C)** The heatmap shows the expression profiles of 10 MES-related lncRNAs between the high- and low-risk groups in the TCGA database. **(D)** Risk score distribution validation in CGGA database. **(E)** Survival status and time of glioma patient validation in CGGA database. **(F)** The heatmap shows the expression profiles of 10 MES-related lncRNAs between the high- and low-risk groups validation in the CGGA database.

**Figure 4:**
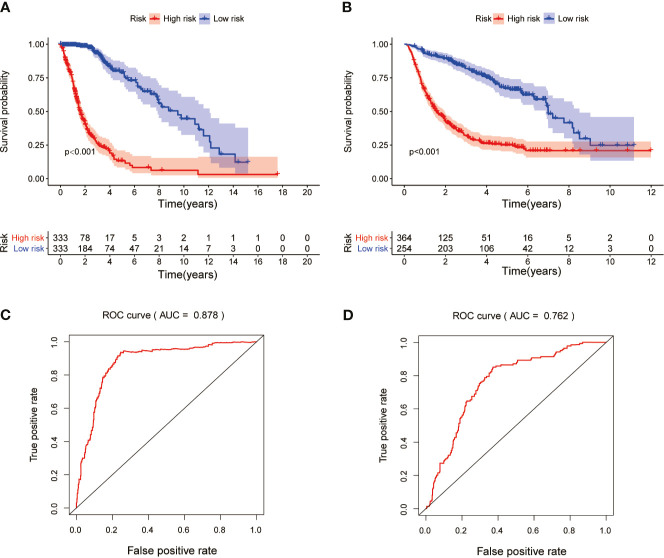
Kaplan-Meier survival and ROC curve analysis in the TCGA and CGGA data sets. **(A)** Kaplan-Meier survival analysis based on 10 MES-related lncRNAs between the high- and low-risk groups in TCGA testing set. **(B)** Kaplan-Meier survival curve for CGGA independent validation set. **(C)** ROC curve shows the performance of the prognostic models in predicting OS of glioma patients from TCGA testing set. **(D)** ROC curve analysis for CGGA independent validation set.

Risk score model was constructed by perform multivariate Cox regression analysis. The risk score of each patient was calculated according to the linear combination of regression coefficients and lncRNA expression values in the TCGA database. Based on the median risk score, the patients from TCGA were separated into high- and low-risk groups (**Figure 3A**); patients with a higher risk score demonstrated lower survival time (**Figure 3B**).The heatmap showed distinct differences in the expression levels of the ten prognostic-related lncRNAs in the low- and high-risk groups (**Figure 3C**). In the TCGA data set, compared with the high-risk group, the overall survival (OS) of glioma patients in the low-risk group was longer (**Figure 4A**). The time-dependent receiver operating characteristic (ROC) curve showed an area under curve (AUC) of 0.878 (**Figure 4C**), indicating that the model provided higher prediction accuracy. In the CGGA validation data set, we also adopted a median risk score to distinguish between high- and low-risk groups (**Figure 3D**). As expected, patients in the database with a higher risk score demonstrated lower survival time and the expression patterns of these lncRNAs were similar to those in the TCGA data set (**Figures 3E, F** **)**. Supplementally, the high-risk group showed poor prognosis in the CGGA dataset, with an AUC of 0.762 (**Figures 4B, D** **)**.

### The Ten MES-Related LncRNAs as a Prognostic Model Is an Independent Factor for Glioma Patients

**Figure 5:**
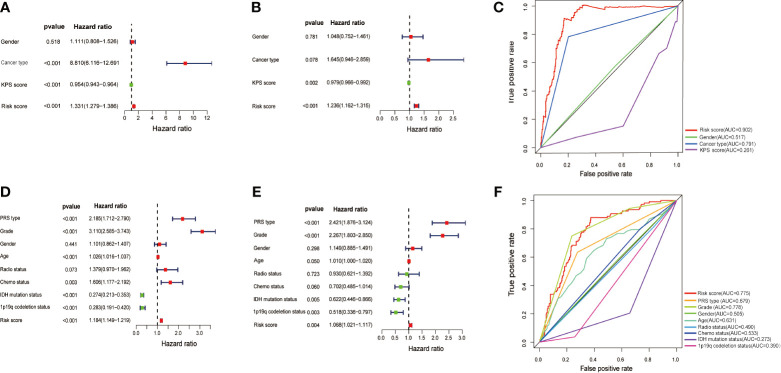
Assessment of the independent prognostic value of the risk score model based on a 10 MES-related lncRNAs signature. **(A)** Univariate and **(B)** multivariate Cox regression analyses of risk score and other clinical factors in the TCGA data set. **(C)** The AUC based on the ROC curves for risk score and other clinical factors in the TCGA data set; Clinical factors: gender, cancer type, KPS score, risk score. **(D)** Univariate and **(E)** multivariate Cox regression analysis of risk score and other clinical factors in the CGGA data set. **(F)** The AUC for risk score and other clinical factors in the CGGA data set; Clinical factors: PRS type, grade. gender, age, radio status, chemo status, IDH mutation status, 1p19q codeletion status, risk score.

To determine whether the prognostic model of the above ten MES-related lncRNAs was an independent factor for glioma patients, we conducted univariate and multivariate Cox regression analyses in with the TCGA dataset. The hazard ratio (HR) of the risk score and 95% CI were 1.331 and 1.279-1.386 (P<0.001) in univariate (**Figure 5A**), and 1.236 and 1.162-1.315 (P<0.001) in multivariate Cox regression analyses (**Figure 5B**), respectively. In order to evaluate the predictive accuracy of the risk score on the prognosis of glioma patients, the AUC of the risk score was calculated to be 0.902, which was more than the AUCs of gender, cancer type, and KPS score (**Figure 5C**). Furthermore, we conducted the same analysis with the CGGA database for verification. The HR of the risk score and 95% CI were 1.184 and 1.149-1.219 (P< 0.001) in univariate (**Figure 5D**), and 1.068 and 1.021-1.117 (P< 0.05) in multivariate Cox regression analyses (**Figure 5E**), respectively. The ROC curve showed that the AUC of the risk score was 0.775, similar to the AUC of grade (0.778) (**Figure 5F**). These results indicate that the ten MES-related lncRNAs signature as a prognostic model is a significant independent prognostic factor for glioma patients.

### PCA and Immune Infiltration in Different Risk Groups

**Figure 6:**
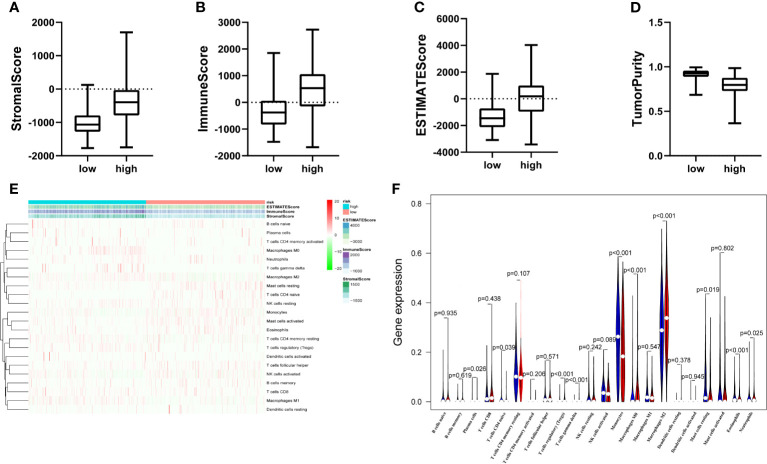
The landscape of immune infiltration and estimating tumor purity. **(A–D)** There is a statistical difference of the stromal score **(A)** immune score **(B)** ESTIMA TE score **(C)** and Tumor Purity **(D)** between the high- and the low-risk groups. **(E)** The heatmap shows the stromal score immune score, ESTIMATE score and corresponding 22 immune cell proportions of each glioma patient in the two risk groups. The horizontal axis shows the samples which were divided into two risk groups. **(F)** The violin plot revealed the distribution of same immune cells between two risk groups. (Blue was low-risk group and red was high-risk group).

Principal component analysis (PCA) showed a distinctive distribution between low- and high-risk groups based on the ten MES-related lncRNAs in TCGA, suggesting that the risk model could divide glioma patients into two parts. The samples in the low- and high-risk groups are represented by green and red dots, respectively in **Figure S2**. MES subtype is closely associated with immune. Next, we calculated the stromal score, immune score, ESTIMA TE score and tumor purity of every glioma patients adopting the ESTIMA TE algorithm. Compared with high-risk group, the box chart showed that the low-risk group was significantly lower in stromal score, immune score and ESTIMA TE score, meanwhile, had higher tumor purity (**Figures 6A–D**). The heatmap and violin plot showed that there were marked differences in the relative proportions of 6 out of 22 immune cells. Among them, T cells regulatory(Tregs), T cells gamma delta, Macrophages M0 and Macrophages M2(all above p < 0.001) presented higher proportions in high-risk group compared with low-risk group, and Monocytes and Eosinophils(all above p < 0.001) were significantly upregulated in the low-risk group **(** **Figures 6E, F** **)**.

### Functional Enrichment Analysis based on the Ten MES-Related LncRNAs Signature

**Figure 7:**
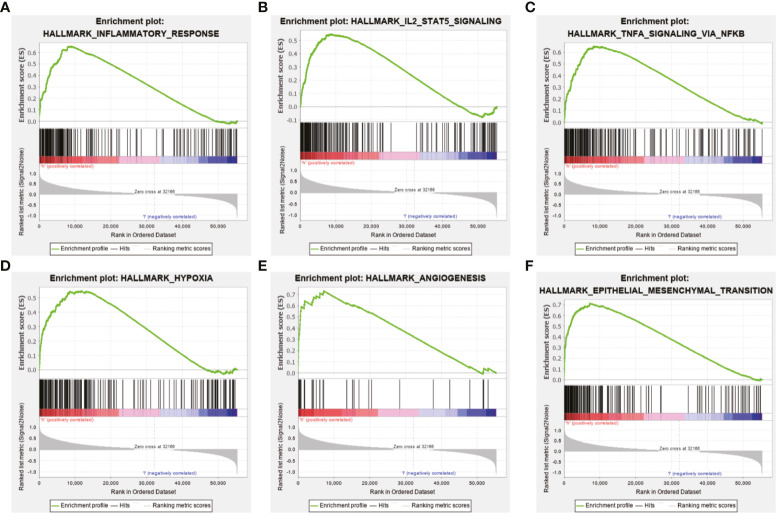
Functional enrichment analysis based on the prognostic model in the TCGA data set. GSEA indicated obvious enrichment of **(A)** inflammatory response, **(B)** IL 2/STAT5 signaling, **(C)** TNFα signaling *via* NF-κB, **(D)** hypoxia, **(E)** angiogenesis, and **(F)** EMT in the high-risk group.

Further functional annotation was conducted using GSEA. In the high-risk group, we discovered that a total of six gene sets were significantly enriched in tumor-related pathways, including inflammatory response, interleukin (IL)2/signal transducer and activator of transcription (STAT) 5 signaling and tumor necrosis factor α (TNFα) signaling *via* nuclear factor-κB (NFκB) were closely associated with tumorigenesis and malignant phenotypes such as migration and invasion of glioma (**Figures 7A–C**). Additionally, hypoxia, angiogenesis, and epithelial mesenchymal transition (EMT) were closely related to the invasion and metastasis of glioma (**Figures 7D–F**). The above results further revealed that the prognosis model based on the ten MES-related lncRNAs may illustrate the underlying mechanism of the occurrence and development of glioma, which leads to a worse prognosis for the patients.

### Knocking Down the Expression of DGCR10, HRA1B, and SNHG18 Can Significantly Impact Glioma Cells Migration and Invasion

**Figure 8:**
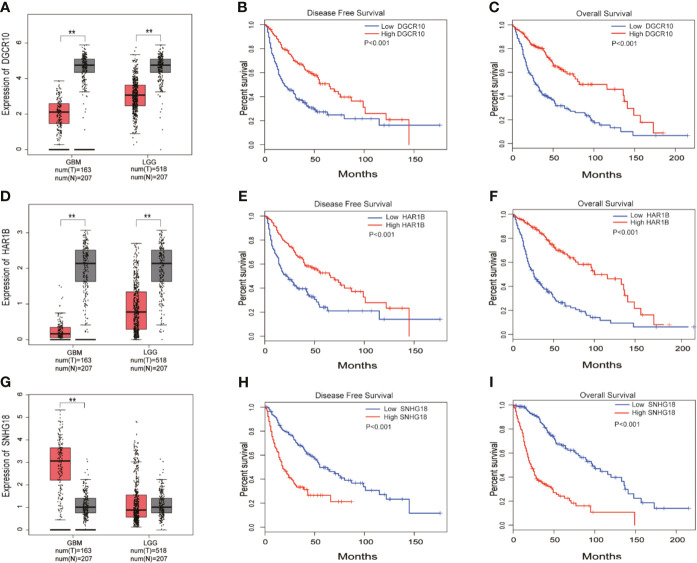
DGCR10, HAR1B, and SNHG18 were selected from the 10 MES-related lncRNAs. **(A)** Differences in DGCR10 expression between the normal and glioma groups from the TCGA and GTEX data sets. **(B)** Disease-free survival analysis of DGCR10 from the TCGA database. **(C)** Overall survival analysis of DGCR10 from the TCGA database. **(D)** Differences in HAR1B expression between the normal and the glioma groups from the TCGA and GTEX data sets. **(E)** Disease-free survival analysis of HAR1B from the TCGA database. **(F)** Overall survival analysis of HAR1B from the TCGA database. **(G)** Differences in SNHG18 expression between the normal and glioma groups from the TCGA and GTEX data sets. **(H)** Disease-free survival analysis of SNHG18 from the TCGA database. **(I)** Overall survival analysis of SNHG18 from the TCGA database. **p < 0.01.

**Figure 9:**
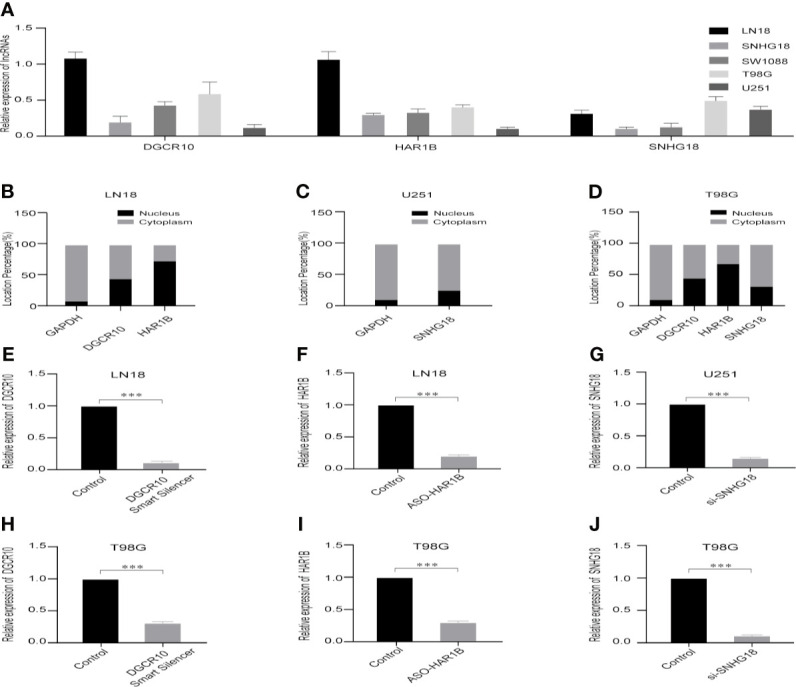
Selection of cell lines and verification of the knockdown effect. **(A)** Relative expression levels of DGCR10, HAR1B, and SNHG18 in five cell lines. **(B)** The nuclear and cytoplasmic percentage of DGCR10 and HAR1B in LN18 cells. **(C)** The nuclear and cytoplasmic percentage of SNHG18 in U251 cells. **(D)** The nuclear and cytoplasmic percentage of DGCR10, HAR1B, and SNHG18 in T98 cells. **(E)** The relative expression level of DGCR10 in LN18 cells after knockdown. **(F)** The relative expression level of HAR1B in LN18 cells after knockdown. **(G)** The relative expression level of SNHG18 in U251 cells after knockdown. **(H)** The relative expression level of DGCR10 in T98G cells after knockdown. **(I)** The relative expression level of HAR1B in T98G cells after knockdown. **(J)** The relative expression level of SNHG18 in T98G cells after knockdown. ***p < 0.001.

**Figure 10:**
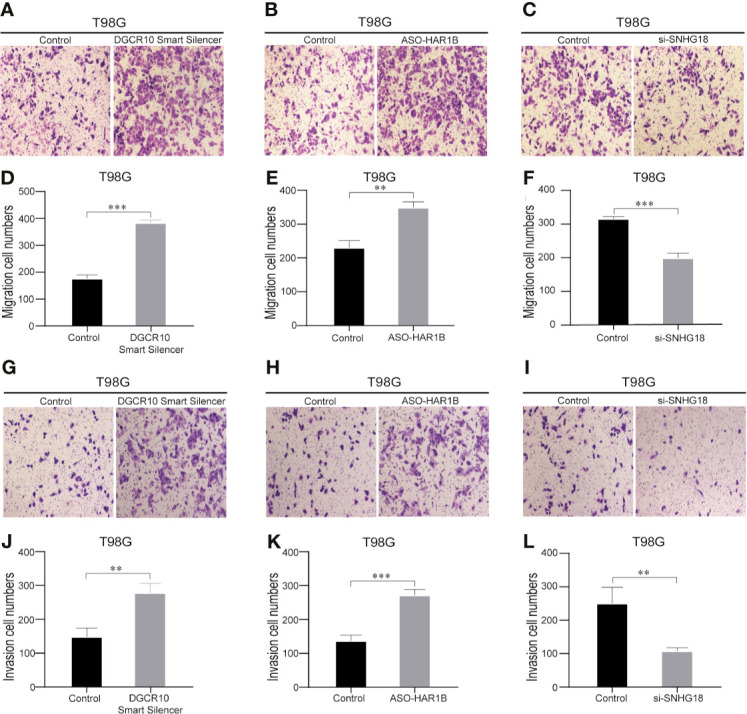
Knockdown of DGCR10, HAR1B and SNHG18 impact the T98G cell migration and invasion ability. Representative imaging **(A–C)** or counting **(D–F)** of migration assays after knockdown of DGCR10, HAR1B, and SNHG18 in T98G cell lines. Scale bar, 200μm. Representative imaging **(G–I)** or counting **(J–L)** of invasion assays after knockdown of DGCR10, HAR1B, and SNHG18 in T98G cell lines. Scale bar, 200μm; **p < 0.01; ***p < 0.001.

Based on the samples from the TCGA and Genotype-Tissue Expression Portal (GTEx) datasets, the RNA sequencing expression data retrieved from the GEPIA website were used to analyze the differential expression level of the ten lncRNAs between the normal group and tumor group of glioma (**Figures 8A, D, G** and **Figure S3**). Given that the functions of DGCR10, HAR1B, and SNHG18 are rarely studied in gliomas, the patients from the TCGA database were classified into high- and low-expression groups according to the median expression level of these three lncRNAs. Disease-free survival and overall survival rates showed that the expression of DGCR10 and HAR1B are positively correlated with the OS of glioma patients (**Figures 8B, C, E, F** **)**. In contrast, the expression levels of SNHG18 are negatively correlated with OS (**Figures 8H, I** **)**. Subsequently, functional studies were carried out in different glioma cell lines. Real-time quantitative PCR (RT-qPCR) was carried out to detect the expression levels of DGCR10, HAR1B, and SNHG18 in five glioma cell lines. It was found that DGCR10 and HAR1B were expressed at a relatively high level in LN18 and T98G, whereas SNHG18 was expressed at a high level in U251 and T98G (**Figure 9A**). Next, nucleocytoplasmic fractionation was performed to determine the locations of DGCR10, HAR1B, and SNHG18 in glioma cells. The results revealed that DGCR10 and SNHG18 were mainly expressed in the cytoplasm, whereas HAR1B were mainly expressed in the nucleus(**Figures 9B–D**). To verify the effects of these lncRNAs on the migration and invasion of glioma cells, we knocked down the expressions of DGCR10, HAR1B, and SNHG18 in glioma cells (**Figures 9E–G** and **Table 3**), and the knockdown efficiency was verified in T98G cells (**Figures 9H–J** and **Table 3**). The results of transwell and matrigel invasion assays showed that T98 cells’ ability to migrate and invade was significantly increased after knockdown of DGCR10 and HAR1B, while the ability was significantly decreased after knockdown of SNHG18 (**Figures 10A–L** and **Table 4**). In addition, we obtained the same results of migration and invasion in LN18 and U251 cell lines (**Figures S4A–L**).

**Table 3:** The relative expression level of DGCR10, HAR1B and SNHG18 in different glioma cell lines after knockdown.

**Cell lines**	**Control**			**DGCR10 Smart Silence**		
LN18	1.00	1.00	1.00	0.09	0.11	0.13
T98G	1.00	1.00	1.00	0.31	0.28	0.34
**Cell lines**	**Control**			**ASO-HAR1B**		
LN18	1.00	1.00	1.00	0.2	0.21	0.19
T98G	1.00	1.00	1.00	0.3	0.28	0.31
**Cell lines**	**Control**			**si-SNHG18**		
U251	1.00	1.00	1.00	0.13	0.15	0.17
T98G	1.00	1.00	1.00	0.11	0.09	0.13

**Table 4:** Representative counting of migration and invasion assays after knockdown of DGCR10, HAR1B, and SNHG18 in different glioma cell lines.

Migration cell numbers in T98G					
**Control**			**DGCR10 Smart Silence**		
170	174	173	370	368	373
Control			ASO-HAR1B		
220	218	216	330	326	332
Control			si-SNHG18		
305	302	301	185	183	186
**Invasion cell numbers in T98G**					
Control			DGCR10 Smart Silence		
145	143	140	262	261	263
Control			ASO-HAR1B		
126	128	130	255	256	253
Control			si-SNHG18		
240	236	242	98	101	99

## Discussion

Glioma, especially glioblastoma, is the most destructive tumor of the human nervous system (29). In recent years, despite the progress in the diagnosis and treatment owing to the infiltration and rapid proliferation of gliomas, the tumor is difficult to cure by surgery alone. The prognosis of patients who relapse after surgery is poor, and the median survival time is only extended by a few months (23, 24, 30). The complexity of glioma is reflected by molecular heterogeneity; molecular subtyping offers better predictions of the development of polymorphisms in glioma, and guides scientific treatment strategies (31). The mesenchymal subtypes are especially malignant as compared to the others (neural, classic, and pre-neural types), and the relapsed GBM is always lethal and usually shows a mesenchymal phenotype (32–35). In addition, mesenchymal tumors express higher levels of angiogenic markers besides higher levels of necrosis (8, 33). The transition from proneural to mesenchymal subtype is closely related to treatment resistance and poor prognosis (36).

Next-generation sequencing technology in a growing number of cancer transcriptomes has revealed thousands of lncRNAs whose aberrant expression is associated with the tumor cell biology function, including cell cycle, proliferation, apoptosis, metastasis, invasion, and migration (37, 38). For example, in colorectal cancer (CRC), LINC00460 increases and adjusts the expression of ANXA2, which is associated with the expression of E-cadherin and N-cadherin, which promote cell invasion and EMT (39). Many lncRNAs are upregulated in gliomas and promote the malignant progression of glioma cells. NEAT1 is an lncRNA confirmed to be upregulated in gliomas and promotes cell migration and invasion, in addition to suppressing apoptosis in glioma cells (40). MES-related lncRNAs such as CRNDE can promote cell proliferation, migration, and invasion (41). Notably, the MES is a more malignant molecular subtype with a higher tendency for relapse, metastasis, and increased vascularity compared with the others (42). The study of molecular characteristics aims to determine new markers for the prognosis of cancer patients and therapeutic targets. In the past decade, new advances in bioinformatics and high-throughput technologies have helped improve our ability to understand the pathogenesis and predict the prognosis of cancer patients besides identifying potential biomarkers (43). For example, recent research has shown that ten autophagy-related lncRNAs possess prognostic value for glioma patients (24).Wang et al. confirmed a prognosis model consisting of nine immune-related lncRNAs in anaplastic glioma patients (44). Given the molecular diversity of glioma and the malignant manifestations of mesenchymal subtypes, it is essential to construct a prognostic model based on molecular characteristics (45).

In this work, we used bioinformatics and statistical tools to systematically analyze the prognostic accuracy of lncRNAs associated with mesenchymal subtype, similar to the construction of immune-related lncRNAs model (46). We integrated multiple MES-related lncRNAs into a single model and explored whether the model played a more important role in the prognostic evaluation of gliomas. First, 303 MES-related genes were obtained from the TCGA and Ivy GAP data sets, and 47 corresponding lncRNAs were acquired by performing co-expression analysis (47). Then, 42 differentially expressed lncRNAs were screened between LGG and GBM samples. Finally, a candidate prognosis model consisting of ten OS-related lncRNAs was constructed by performing univariate and multivariate Cox analyses. The accuracy and predictability of the model were tested and verified in TCGA and CGGA databases. The results showed that the risk score model could accurately predicted the prognosis of glioma. In clinical work, risk scores of glioma patients can be calculated based on the regression coefficients and the expression values of the ten MES-related lncRNAs, and determine whether the patient is low- or high-risk group and predict their prognosis.

Increasing evidence indicated that immune checkpoint receptor target was highly enriched in mesenchymal subtype glioma and might be a potential marker of mesenchymal subtype (17, 18). Moreover, research shows that Tumor-infiltrating immune cells (TIICs) play diverse roles in glioma and low tumor purity is related to unfavorable prognosis in glioma (48–50). Our ESTIMA TE algorithm showed high-risk group had higher stromal score, immune score, ESTIMA TE score and lower tumor purity. Regulatory T-cells (Tregs) are immunosuppressive T-cells that normally prevent autoimmunity when the human immune response is evoked. In addition, hypoxia is characteristic of tumor development and is also correlated with induction of Tregs (51). The phenotype of glioma-associated macrophages might be quite different from the other malignant solid tumors and is prone to M0-like phenotype (52). Study indicates that M2-like macrophages drove glioma Vasculogenic mimicry (VM) through amplifying IL-6 secretion in glioma cells via PKC pathway (53). Monocytes bridge innate and adaptive immune responses and can affect the tumor microenvironment through give rise to antitumor effectors and activate antigen-presenting cells (54). A negative correlation between peripheral eosinophils and glioma grade was found in one study. Numerous cytokines derived from eosinophils could regulate the immune response and affect the tumor microenvironment (55). Consistent with above conclusions, the landscape of immune infiltration indicated that T cells regulatory(Tregs), Macrophages M0 and Macrophages M2 presented higher proportions in high-risk group compared with low-risk group. The relative proportions of Monocytes and Eosinophils were significantly upregulated in the low-risk group. These results suggest that the heterogeneity of TIICs in gliomas is evident and may play a role in the malignant progression of glioma.

Next, we further explored the potential mechanisms by functional analysis. Inflammatory factors such as TNF‐α was reported to be significantly associated with the malignant progression of glioma cells and sustained activation of NFκB, which is caused by TNFα, leading to neuroblastoma recrudescence and regulation of cell invasion and metastasis (56, 57). As an inflammatory mediator, STAT5 also promotes motility and proliferation of glioma cells (58). In addition, hypoxia acquires the process of carcinogenesis and angiogenesis, which leads to the migration and invasion of glioma cell lines, and angiogenesis significantly worsens prognosis of cancer patients (59–62). Notably, hypoxia has been shown to promote EMT, which is crucial for malignant progression of tumors (63–65). Consistent with these studies, the results of GSEA showed that the high-risk group was enriched in the inflammation-related pathways and malignant biological processes such as hypoxia, angiogenesis, and EMT. Considering that the high-risk group is closely related to the malignant progression of tumors, especially the migration and invasion of cells, we selected the protective factors DGCR10 and HAR1B, and risk factor SNHG18 for migration and invasion experiments. Compared with other lncRNAs in our prognostic model, the effect of these three lncRNAs on the function of glioma cells is rarely reported. Overexpression of DGCR10 inhibits non-small cell lung cancer (NSCLC) cell migration and invasion. Moreover, DGCR10 acts as a tumor suppressor *via* sequestering miR-2861 in papillary thyroid carcinoma (66, 67). Research has confirmed that elevated expression of HAR1B was significant for better OS in hepatocellular carcinoma (68). High expression of SNHG18 may be a marker of poor prognosis in multiple myeloma (MM) (69). Similar to the function of these lncRNAs reported in other types of tumors, in our study, knockdown of DGCR10 and HAR1B promoted, whereas knockdown of SNHG18 inhibited the migration and invasion of glioma cells.

In summary, we screened ten MES-related lncRNAs and classified low- and high-risk groups based on the median risk score, which can be used to identify glioma patients with poor prognosis. Further GSEA and functional experiments confirmed that DGCR10, HAR1B, and SNHG18 can be potentially used as personalized biomarkers to predict treatment outcomes. In recent years, there have been many studies based on the generation of models for prediction of prognosis for glioma patients, but there have been no similar studies using MES-related lncRNAs as a prognostic model (70). We believe that these ten MES-related lncRNAs are potential prognostic markers, which will provide a good reference for cancer researchers. In order to complete a more comprehensive research, focus on the following points is required in the future. First, other well-known clinical prognostic factors that could not be obtained from the database should be the focus of research. Second, in-depth studies of the 10 MES-related lncRNAs, such as molecular mechanisms and animal experiments, are needed.

## Data Availability Statement

The original contributions presented in the study are included in the article/**Supplementary Material**. Further inquiries can be directed to the corresponding authors.

## Author Contributions

BZ and EB designed this article and provided funding support. KH and XY were responsible for software analysis and conducting experience. YZ and ZZ was responsible for collating the data and recording the experience results. MC and LL was in charge of article figure. ZC and ZY drafted the article. All authors contributed to the article and approved the submitted version.

## Funding

This project was supported by the National Natural Science Foundation of China (No. 81972348), Key Research and Development Plan Project of Anhui Province (No.1804h08020270), College Excellent Youth Talent Support Program in Anhui Province (No.gxypZD2019019), Key Projects of Natural Science Research in Anhui Province (KJ2019A0267), Academic Funding Project for Top Talents in Colleges and Universities in Anhui Province (No. gxbjZD10), Nova Pew Plan of the Second Affiliated Hospital of Anhui Medical University (No.2017KA01). Open Projects of Key Laboratory in Medical Physics and Technology of Anhui Province (LHJJ202001).

## Conflict of Interest

The authors declare that the research was conducted in the absence of any commercial or financial relationships that could be construed as a potential conflict of interest.

## Publisher’s Note

All claims expressed in this article are solely those of the authors and do not necessarily represent those of their affiliated organizations, or those of the publisher, the editors and the reviewers. Any product that may be evaluated in this article, or claim that may be made by its manufacturer, is not guaranteed or endorsed by the publisher.

## Supplementary Material

Supplementary Figure 1 Construction of MES-related lncRNAs prognostic models in glioma patients. **(A)** The forest plot showing the candidate 18 MES-related lncRNAs correlated with OS by univariate Cox analysis in the TCGA data set. **(B)** Multivariate Cox analysis showing the 10 MES-related lncRNAs correlated with OS in the TCGA data set.

Supplementary Figure 2 PCA between low- and high- risk groups based on the 10 MES-related lncRNAs expression profiles.

Supplementary Figure 3 The relative expression of the remaining 7 lncRNAs contained in the prognostic model. **(A)** Differences in GDNF-AS1 expression between the normal and glioma groups from the TCGA and GTEX data sets. **(B)** Differences in CRNDE expression between the normal and glioma groups from the TCGA and GTEX data sets. **(C)** Differences in FAM201A expression between the normal and glioma groups from the TCGA and GTEX data sets. **(D)** Differences in AGAP2-AS1 expression between the normal and glioma groups from the TCGA and GTEX data sets. **(E)** Differences in RNF219-AS1 expression between the normal and glioma groups from the TCGA and GTEX data sets. **(F)** Differences in LINC00906 expression between the normal and glioma groups from the TCGA and GTEX data sets. **(G)** Differences in HAR1A expression between the normal and glioma groups from the TCGA and GTEX data sets. **p < 0.01; ***p < 0.001.

Supplementary Figure 4 Knockdown of DGCR10, HAR1B, and SNHG18 impact the LN18 and U251 cell migration and invasion ability. Representative imaging **(A, B)** or counting **(D, E)** of migration assays after knockdown of DGCR10 and HAR1B in LN18 cell lines. Representative imaging **(C)** or counting **(F)** of migration assays after knockdown of SNHG18 in U251 cell lines. Representative imaging **(G, H)** or counting **(J, K)** of invasion assays after knockdown of DGCR10 and HAR1B in LN18 cell lines. Representative imaging **(I)** or counting **(L)** of invasion assays after knockdown of SNHG18 in U251 cell lines. Scale bar, 200μm; **p < 0.01; ***p < 0.001.
