# Supplementary figures and images for: Development and Validation of an Mesenchymal-Related Long Non-Coding RNA Prognostic Model in Glioma
Source: Front Oncol. 2021 Sep 3;11:726745. doi: 10.3389/fonc.2021.726745 (PMC8446619; doi:10.3389/fonc.2021.726745)

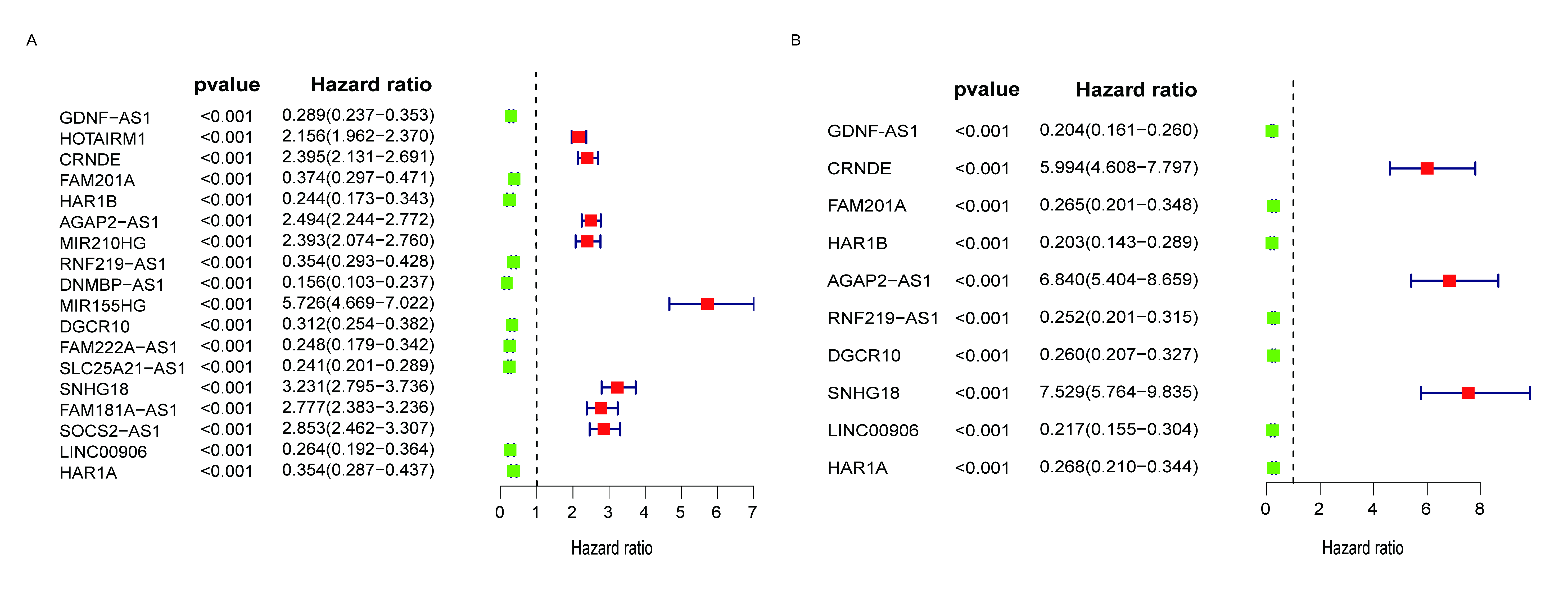

Supplement: Supplementary Figure 1 — Construction of MES-related lncRNAs prognostic models in glioma patients. (A) The forest plot showing the candidate 18 MES-related lncRNAs correlated with OS by univariate Cox analysis in the TCGA data set. (B) Multivariate Cox analysis showing the 10 MES-related lncRNAs correlated with OS in the TCGA data set. [file Image_1.tif]

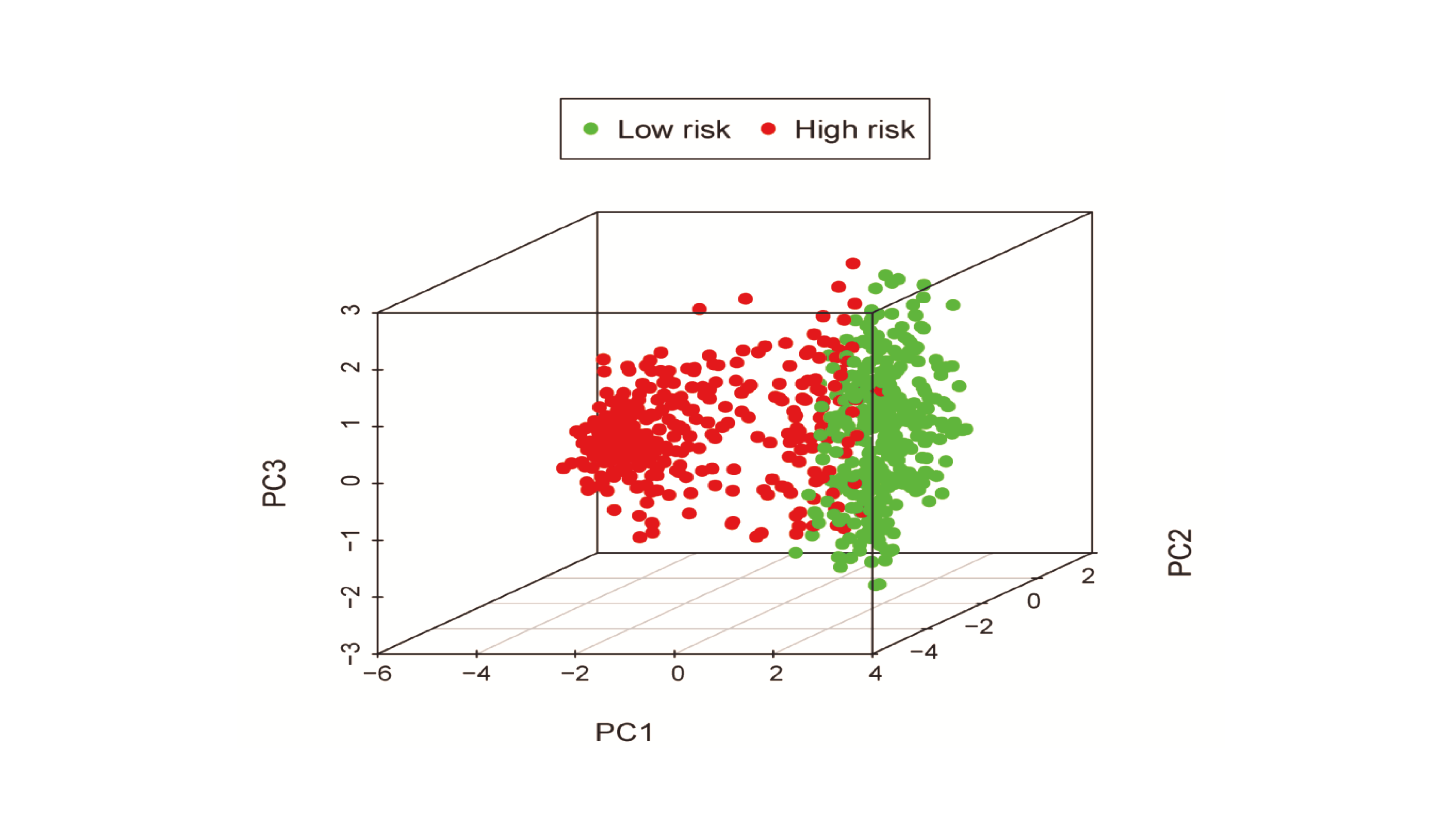

Supplement: Supplementary Figure 2 — PCA between low- and high- risk groups based on the 10 MES-related lncRNAs expression profiles. [file Image_2.tif]

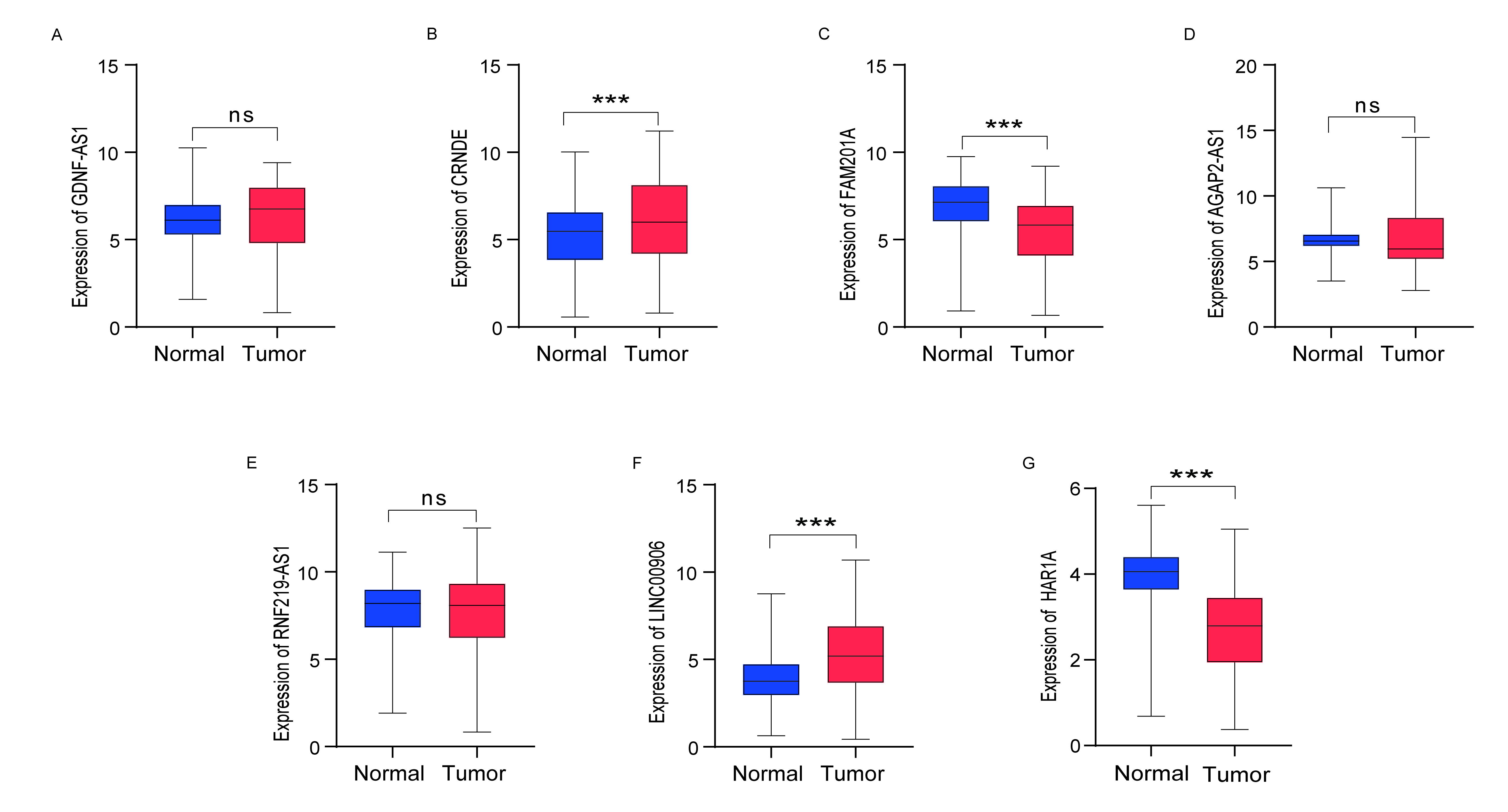

Supplement: Supplementary Figure 3 — The relative expression of the remaining 7 lncRNAs contained in the prognostic model. (A) Differences in GDNF-AS1 expression between the normal and glioma groups from the TCGA and GTEX data sets. (B) Differences in CRNDE expression between the normal and glioma groups from the TCGA and GTEX data sets. (C) Differences in FAM201A expression between the normal and glioma groups from the TCGA and GTEX data sets. (D) Differences in AGAP2-AS1 expression between the normal and glioma groups from the TCGA and GTEX data sets. (E) Differences in RNF219-AS1 expression between the normal and glioma groups from the TCGA and GTEX data sets. (F) Differences in LINC00906 expression between the normal and glioma groups from the TCGA and GTEX data sets. (G) Differences in HAR1A expression between the normal and glioma groups from the TCGA and GTEX data sets. **p < 0.01; ***p < 0.001. [file Image_3.tif]

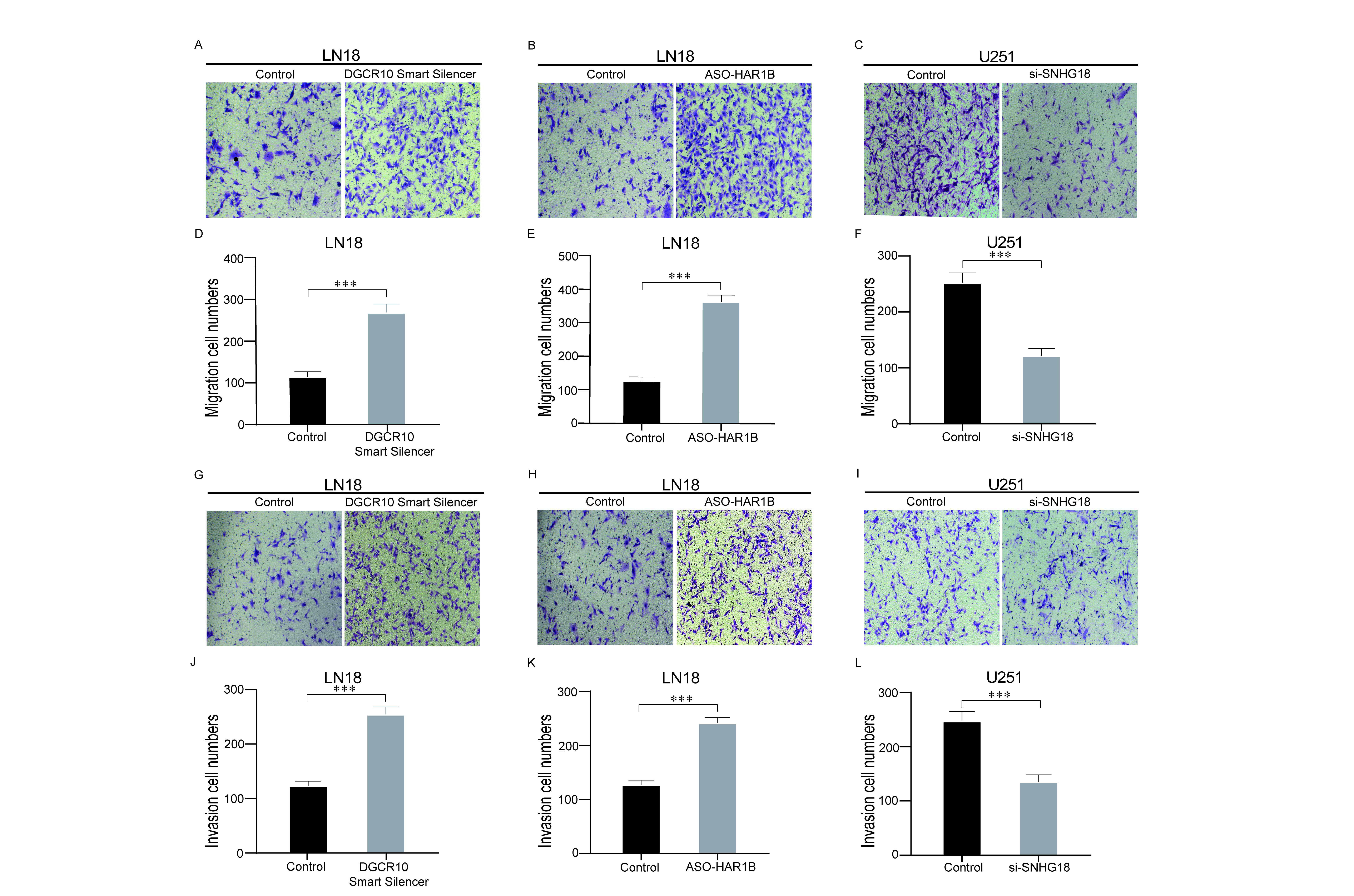

Supplement: Supplementary Figure 4 — Knockdown of DGCR10, HAR1B, and SNHG18 impact the LN18 and U251 cell migration and invasion ability. Representative imaging (A, B) or counting (D, E) of migration assays after knockdown of DGCR10 and HAR1B in LN18 cell lines. Representative imaging (C) or counting (F) of migration assays after knockdown of SNHG18 in U251 cell lines. Representative imaging (G, H) or counting (J, K) of invasion assays after knockdown of DGCR10 and HAR1B in LN18 cell lines. Representative imaging (I) or counting (L) of invasion assays after knockdown of SNHG18 in U251 cell lines. Scale bar, 200μm; **p < 0.01; ***p < 0.001. [file Image_4.tif]
